# Maternal Body Mass Index, Gestational Weight Gain, and Risk of Cancer in Offspring: A Systematic Review and Meta-Analysis

**DOI:** 10.3390/nu15071601

**Published:** 2023-03-25

**Authors:** Junxiang Miao, Yan Chen, Xiaoling Liu, Changxiang Ye, Xuan Zhou, Ziqi Yang, Ziqiang Gong, Lizhang Chen, Tingting Wang

**Affiliations:** 1 Department of Epidemiology and Health Statistics, Xiangya School of Public Health, Central South University, Changsha 410017, China; 2 Hunan Provincial Key Laboratory of Clinical Epidemiology, Changsha 410078, China; 3 NHC Key Laboratory for Birth Defect for Research and Prevention, Hunan Provincial Maternal and Child Health Care Hospital, Changsha 410007, China

**Keywords:** gestational weight gain, BMI, cancer, meta-analysis

## Abstract

**Background**: Mounting evidence suggests that maternal obesity and gestational weight gain (GWG) may increase the risk of cancer in their offspring; however, results are inconsistent. The purpose of this research is to determine the association between maternal body mass index (BMI) and GWG and the risk of cancer in offspring through a systematic and comprehensive meta-analysis. **Methods**: A systematic literature search of several databases was conducted on 1 October 2022 to identify relevant studies. The quality of the included studies was evaluated using the Newcastle–Ottawa scale. The overall risk estimates were pooled using a random-effects meta-analysis. **Results**: Twenty-two studies with more than 8 million participants were included. An increased risk of total cancer was found in offspring whose mothers had a high GWG (odds ratio [OR]: 1.10; 95% CI: 1.01–1.19; *p*: 0.040) but not in offspring whose mothers had a low GWG (OR: 1.06; 95% CI: 0.96–1.17; *p*: 0.030), when compared with offspring whose mothers had a suitable GWG. In addition, no statistically significant association was found between maternal underweight (OR: 1.05; 95% CI: 0.97–1.13; *p*: 0.630), overweight/obesity (OR: 1.07; 95% CI: 0.99–1.16; *p*: 0.020), and risk of total cancer in offspring. **Conclusions**: Our study proposes evidence that maternal BMI and GWG may be associated with the risk of cancer in offspring, although statistical significance was found only for high GWG. Further well-designed research is required to clarify the potential relevance of maternal BMI and GWG on offspring cancer, especially for specific cancers.

## 1. Introduction

As a common cause of death worldwide, cancer brings increasing health and economic burden. It has become a persistent public health challenge and an important obstacle to the increase in human life expectancy [1,2]. In recent years, new cancer cases and deaths are increasing every year. The latest data show that the global cancer burden is as high as 19.3 million new cases and 10.0 million deaths in 2020 [3]. Therefore, it is imperative to identify cancer risk factors and target prevention in high-risk populations, which will be a benefit to improving global health.

Studies have shown that factors such as drinking, smoking, and being overweight/obese contribute to a higher risk of cancer [4,5,6]. The association and possible mechanisms of self-overweight and obesity in promoting self-cancer have been extensively studied by many researchers [7,8,9,10,11,12]. Furthermore, the potential relevance of maternal obesity on cancer in offspring has attracted more and more attention over the past few years. Mounting evidence suggests that maternal obesity may increase the risk of cancer in offspring. Meanwhile, a possible link is also observed between maternal gestational weight gain (GWG) and cancer risk in offspring [13,14,15,16]. Considering the relative prevalence of overweight/obesity in pregnant women (the prevalence of overweight/obesity women during pregnancy ranges from 12.3% to 63.5%) and the serious financial and health burden of cancer, even a small risk can lead to a severe disease burden [17,18,19]. However, the available evidence regarding maternal overweight/obesity and GWG and the risk of cancer in offspring is inconsistent [14,16,20,21,22,23]. In this situation, the use of comprehensive methods (for example, meta-analysis) to evaluate the data provided in scientific studies will help to clarify the relationship between maternal body mass index (BMI) and GWG and offspring cancer.

So far, only one relevant meta-analysis published in 2010 focused on the relationship between maternal BMI and risk of testicular cancer in male offspring [24], in which no significant association was found. However, this meta-analysis was not exhaustive because the risks of other major cancers were not reported, such as leukemia, brain cancer, and breast cancer. In addition, several studies with large samples have been published after this meta-analysis. Therefore, it is necessary to resummarize the available evidence to determine the association between maternal BMI and offspring cancer.

To this end, we aim to perform a systematic and comprehensive meta-analysis regarding the relationship between maternal BMI and the risk of offspring cancer. Our study also quantitatively summarized present evidence on the association between maternal GWG and offspring cancer risk since there was still no relevant meta-analysis. Our meta-analysis may help to access the risk of cancer in offspring associated with maternal factors.

## 2. Methods

### 2.1. Search Strategy

This meta-analysis was presented following Preferred Reporting Items for Systematic Reviews and Meta-Analysis (PRISMA) statement and Meta-analysis of Observational Studies in Epidemiology (MOOSE) reporting guidelines [25,26]. Web of Science, PubMed, and Embase were systematically searched by two authors on 1 October 2022. The search terms were as follows: (1) BMI, body mass index, obese, obesity, overweight, and weight; (2) weight gain, change, increase, trajector*, and growth; (3) maternal, perinatal, pregnancy*, trimester, gestational, gestation*, pregnant, conception, gravidity, pre-pregnancy, prepregnancy, pre-conception, antenatal, and prenatal; (4) cancer, tumor*, tumor*, melanoma, neoplasm*, phyma*, nub*, retinoblastoma, lymphoma, leukemia, neuroblastoma, extraosseous sarcomas, and hematological malignancy*. Reference lists of all selected literature were searched to identify further relevant literature.

### 2.2. Exposure and Outcomes

The exposure of interest was maternal BMI and GWG. BMI is classified into the following four groups: underweight (BMI < 18.5 kg/m^2^), normal weight (BMI: 18.5–25 kg/m^2^), overweight (BMI: 25–30 kg/m^2^), and obesity (BMI > 30 kg/m^2^). GWG categories included low GWG (<10 kg or inadequate GWG according to 2009 the Institute of Medicine [IOM] guidelines), normal GWG (10–15 kg or adequate GWG according to 2009 IOM guidelines), and high GWG (>15 kg or excessive GWG according to 2009 IOM guidelines) [27]. Outcomes of interest were any cancer in offspring, including leukemia, testicular germ-cell cancer, brain cancer, hepatoblastoma, breast cancer, lymphoma, neuroblastoma, retinoblastoma, rhabdomyosarcoma, Wilm’s tumor, etc.

### 2.3. Eligibility Criteria

Studies were considered eligible if they: (1) were published in English; (2) had a case-control or prospective cohort design; (3) had maternal BMI and/or GWG as the exposure was clearly reported; (4) had use of any cancer in offspring; and (5) reported relative risks (RRs), odds ratios (ORs) and hazard ratios (HRs), with corresponding 95% confidence intervals (CIs) to calculate them. The following studies were excluded: (1) letters, case reports, meeting abstracts, or reviews; (2) redundant publications; or (3) studies with unclear or incomplete data. If two or more studies were from the same population, the most comprehensive or latest one was selected.

### 2.4. Data Extraction

All studies obtained through the search strategies were evaluated by two reviewers (MJX and CY) independently. Any differences of opinion were settled through discussion, and if necessary, the third reviewer (WTT) was invited to have the final vote. Data collection was performed by using a self-made data extraction table to evaluate and extract the following data for each included piece of literature: the first author and year of publication, geographic region, study design, sample size, study population, age of participants, ascertainment of maternal weight, maternal weight categories, outcomes reported, confounds adjusted, and risk estimates with corresponding 95% CIs.

### 2.5. Study Quality Assessments

The study quality was assessed independently by two reviewers (MJX and CY), with the Newcastle–Ottawa Scale (NOS) for observational studies [28]. The NOS includes eight items, and the total score is nine.

### 2.6. Statistical Analyses

Relative risks (RRs) were used to measure the association between maternal BMI/GWG and cancer in offspring. RRs were considered odds ratios (ORs) because of the low incidence of cancer in offspring [29]. According to the previously published study, we used both the hazard ratios and the odds ratios to approximate the relative risk [30,31,32]. For studies that reported two or more kinds of cancer, the pooled OR for the total cancer was calculated using a fixed-effect model within each study. About each specific cancer, the pooled estimate was calculated only when there were two or more relevant studies available.

All analyses were conducted using R version 4.0.3 (The R Foundation for Statistical Computing) and RevMan version 5.3 (The Nordic Cochrane Center, Cochrane Collaboration, Copenhagen, Denmark). The pooled ORs and 95% CIs were computed using a random-effects meta-analysis. The heterogeneity of ORs across studies was assessed using the Cochran Q test and the I^2^ statistic. The Cochran Q test was used to evaluate whether the variation across studies was compatible with chance, and *p* < 0.1 was considered to indicate significant heterogeneity. The I^2^ statistic was a quantitative indicator used to evaluate the percentage of the total variance in prevalence estimates due to statistical heterogeneity rather than chance, or sampling error (I^2^ > 75% indicated high heterogeneity, 51–75% indicated substantial heterogeneity, 26–50% indicated moderate heterogeneity, and ≤25% indicated low heterogeneity). To explore the sources of heterogeneity, subgroup analyses were conducted based on different categories: maternal BMI (underweight, overweight/obesity), geographic region (e.g., America, Sweden, the United Kingdom, Israel, France, Australia, Canada), study design (case-control study, cohort study), study population (children, adults), ascertainment of maternal weight (self-reported, medical record), and whether confounding factors were adjusted (yes, no). To assess the robustness of the meta-analysis results, sensitivity analysis was conducted by repeating the meta-analysis after excluding each included study. Begg’s test was used to assess the publication bias. Considering the limited number of included studies, subgroup analyses, sensitivity analysis, and Begg’s test were not performed for specific cancers.

## 3. Results

### 3.1. Identification and Characteristics of Studies

**Figure 1:**
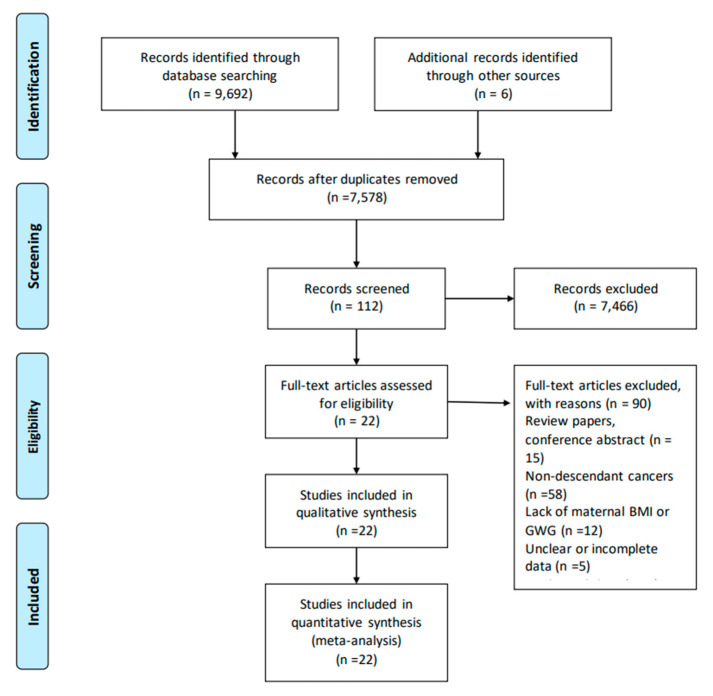
Flow diagram of search strategy and study exclusion with specific reasons.

A total of 7578 records were identified after retrieval, of which 7466 were excluded through the screening of titles and abstracts. Based on a review of the full texts of 112 studies, 90 studies were excluded, mainly because they were reviews, nondescendant cancers, lack of information on maternal BMI or GWG, unclear or incomplete data, or duplicated data. Finally, twenty-two studies were identified as eligible and included in the present meta-analysis (Figure 1) [13,14,15,16,20,21,22,23,33,34,35,36,37,38,39,40,41,42,43,44,45,46].

The characteristics of twenty-two studies with 8,329,446 participants are summarized in Table 1. The included studies were published between 1998 and 2022, including ten cohort studies and twelve case-control studies. Thirteen studies were conducted in America, three in Sweden, and one each in Israel, the United Kingdom, France, Australia, Canada, and Nordic countries, respectively. Fourteen studies evaluated the outcomes in children, five in adults, and three in young adults or adults. Eleven studies used medical records to collect data on maternal BMI and GWG, while eleven were self-reported. Among the twenty-two studies, nineteen studies provided data on maternal BMI, and thirteen studies provided data on GWG. With regard to specific cancers, six studies reported on leukemia, six on testicular germ-cell cancer, four on brain cancer, three on hepatoblastoma, two on breast cancer, two on lymphoma, two on neuroblastoma, and one each on retinoblastoma, colorectal cancer, rhabdomyosarcoma, and Wilms tumor. Confounding factors such as region, age, education, birth weight, and birth order were controlled in nineteen studies. The quality of all studies included here ranged between six and nine scores.

### 3.2. Meta-Analyses of Maternal BMI and Risk of Cancer in Offspring

#### Maternal Underweight and Risk of Cancer in Offspring

**Figure 2:**
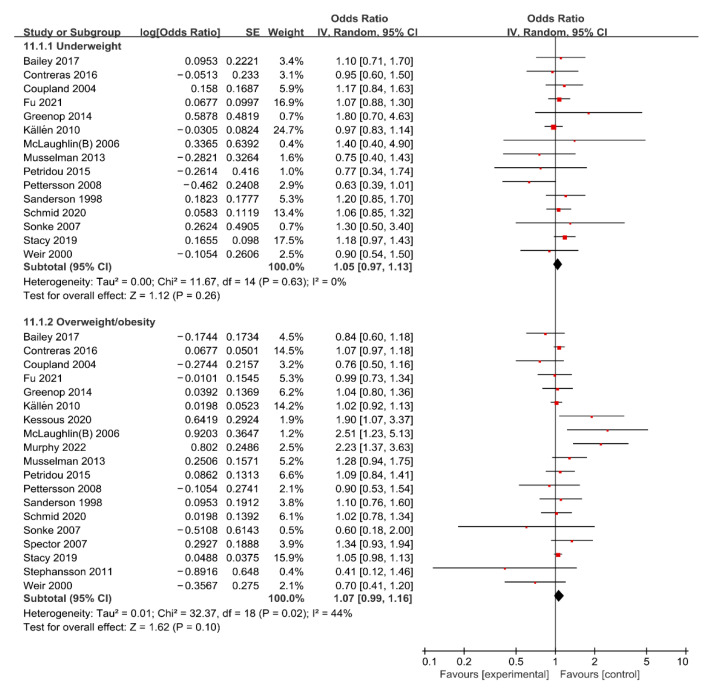
Forest plot of the association between maternal BMI and the risk of total cancer in offspring [13,14,15,16,20,21,22,23,33,34,35,36,38,39,40,42,43,44,45,46].

For maternal underweight, the forest plot of study outcomes is shown in Figure 2. The overall analysis demonstrated that no statistically significant association between maternal underweight and the risk of total cancer in offspring was found (OR: 1.05; 95% CI: 0.97–1.13), with no heterogeneity (I^2^: 0%, *p*: 0.630). Begg’s test found no potential publication bias (z: −0.25, *p*: 0.805). The results of sensitivity analysis suggested that excluding any single research study did not substantially change the overall risk estimates for total cancer, with a range between 1.02 to 1.07 (Supplemental Table S1). Subgroup analyses results of the association between maternal underweight and the risk of total cancer are shown in Table 2. The results of subgroup analyses showed that the variables including geographic region, study design, study population, ascertainment of maternal weight, and whether confounding factors were adjusted were not shown to be associated with the heterogeneity across studies (χ^2^ range: 0.00–1.67, all *p* > 0.05).

### 3.3. Maternal Overweight/Obesity and Risk of Cancer in Offspring

For maternal overweight/obesity, the forest plot of study outcomes is shown in Figure 2. The overall analysis showed that no statistically significant association between maternal overweight/obesity and risk of total cancer was found (OR: 1.07; 95% CI: 0.99–1.16), with substantial heterogeneity (I^2^: 44%, *p*: 0.020). Begg’s test found no potential publication bias (z: −0.45, *p*: 0.649). Results of the sensitivity analysis suggested that excluding any single research did not substantially change the overall risk estimates for total APOs, with a range between 1.05 to 1.08 (Supplemental Table S1). Subgroup analyses results of the association between maternal overweight/obesity and risk of total cancer are shown in Table 2. The results of subgroup analyses showed that the variables including maternal BMI, geographic region, study design, study population, ascertainment of maternal weight, and whether confounding factors were adjusted were not shown to be associated with the heterogeneity across studies (χ^2^ range: 0.02–2.94, all *p* > 0.05).

Risk estimates between maternal overweight/obesity and the risk of specific cancers are summarized in Supplemental Figure S2. The overall analysis suggested that maternal overweight/obesity was associated with a higher risk of leukemia (OR: 1.18, 95% CI: 1.07–1.30) and a lower risk of testicular germ-cell cancer (OR: 0.78, 95% CI: 0.62–0.99), but not associated with risk of brain cancer (OR: 0.94, 95% CI: 0.69–1.29), hepatoblastoma (OR: 1.31, 95% CI: 0.83–2.08), or breast cancer (OR: 1.05, 95% CI: 0.84–1.31) in offspring.

### 3.4. Meta-Analysis of Gestational Weight Gain and Risk of Cancer in Offspring

#### 3.4.1. Maternal Low GWG and Risk of Cancer in Offspring

**Figure 3:**
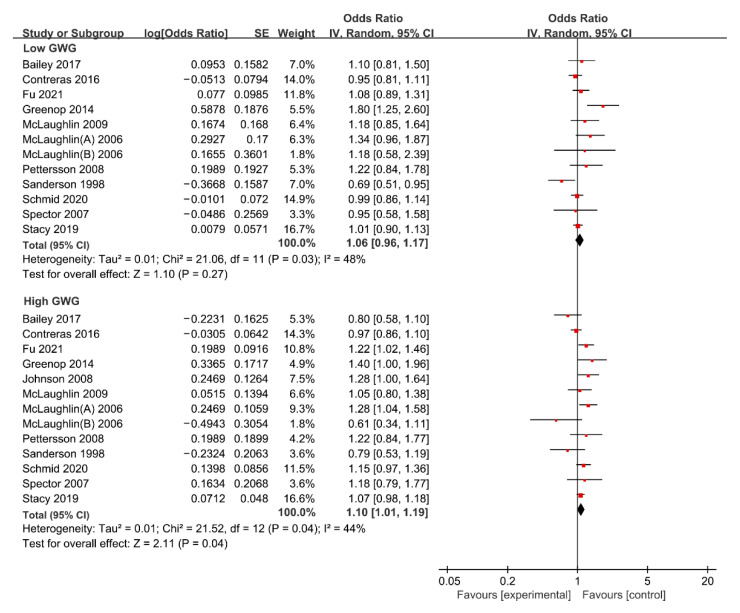
Forest plot of the association between maternal GWG and the risk of total cancer in offspring [13,15,16,20,21,22,33,37,38,39,41,42,45]. GWG, gestational weight gain.

For low GWG, the forest plot of study outcomes is shown in Figure 3. The overall analysis showed that no statistically significant association between low GWG and risk of total cancer was found (OR: 1.06; 95% CI: 0.96–1.17), with substantial heterogeneity (I^2^: 48%, *p*: 0.030). Begg’s test found no potential publication bias (z: 1.37, *p*: 0.170). The results of the sensitivity analysis suggested that excluding any single research study did not substantially change the overall risk estimates for total APOs, with a range between 1.02 to 1.08 (Supplemental Table S1). Subgroup analyses results of the association between low GWG and risk of total cancer are shown in Table 3. The results of subgroup analyses showed that the variables including geographic region, study design, study population, ascertainment of maternal weight, whether confounding factors were adjusted, and whether GWG was classified according to 2009 IOM guidelines were not shown to be associated with the heterogeneity across studies (χ^2^ range: 0.02–3.20, all *p* > 0.05).

Risk estimates between maternal low GWG and risk of specific cancers including leukemia, brain cancer, breast cancer, testicular germ-cell cancer, hepatoblastoma, and neuroblastoma are summarized in Supplemental Figure S3. The overall analysis suggested that maternal underweight was not associated with the risk of leukemia (OR: 1.02, 95% CI: 0.92–1.14), brain cancer (OR: 1.30, 95% CI: 0.91–1.85), breast cancer (OR: 0.85, 95% CI: 0.60–1.20), testicular germ-cell cancer (OR: 1.24, 95% CI: 0.90–1.72), hepatoblastoma (OR: 1.00, 95% CI: 0.61–1.65) or neuroblastoma (OR: 0.99, 95% CI: 0.68–1.45) in offspring.

#### 3.4.2. Maternal High GWG and Risk of Cancer in Offspring

For high GWG, the forest plot of study outcomes is shown in Figure 3. The overall analysis suggested that high GWG significantly increases the risk of total cancer (OR: 1.10; 95% CI: 1.01–1.19), with substantial heterogeneity (I^2^: 44%, *p*: 0.040). Begg’s test found no potential publication bias (z: −0.37, *p*: 0.714). The results of sensitivity analysis suggested that excluding any single research study did not substantially change the overall risk estimates for total APOs, with a range between 1.08 to 1.12 (Supplemental Table S1). Subgroup analyses results of the association between high GWG and the risk of total cancer are shown in Table 3. The results of subgroup analyses showed that the variables including geographic region, study design, study population, ascertainment of maternal weight, whether confounding factors were adjusted, and whether GWG was classified according to 2009 IOM guidelines were not shown to be associated with the heterogeneity across studies (χ^2^ range: 0.00–1.32, all *p* > 0.05).

Risk estimates between maternal high GWG and risk of specific cancers including leukemia, brain cancer, breast cancer, testicular germ-cell cancer, hepatoblastoma, and neuroblastoma are summarized in Supplemental Figure S3. The overall analysis suggested that maternal underweight was not associated with the risk of leukemia (OR: 1.11, 95% CI: 0.97–1.27), brain cancer (OR: 0.93, 95% CI: 0.60–1.45), breast cancer (OR: 1.00, 95% CI: 0.70–1.42), testicular germ-cell cancer (OR: 1.18, 95% CI: 0.86–1.61), hepatoblastoma (OR: 0.88, 95% CI: 0.44–1.78), or neuroblastoma (OR: 1.03, 95% CI: 0.84–1.26) in offspring.

## 4. Discussion

In this meta-analysis, by combining the results of all available cohort and case-control studies with the method of meta-analysis, we provided evidence that maternal high GWG is associated with an increased risk of total cancer with a relative risk estimate of 1.1. Subgroup analysis showed that maternal overweight/obesity was associated with a higher risk of leukemia and a lower risk of testicular germ-cell cancer while no increase in risk was detected for the other subtypes of offspring cancer. As far as we know, this study is the latest comprehensive meta-analysis evaluating the impact of maternal BMI and GWG on the risk of cancer in offspring. Our results may provide valuable and helpful information for women planning pregnancy, pregnant women, and prenatal care providers, and provide another new idea for the primary prevention of cancer.

No statistically significant association between maternal BMI and overall cancer in offspring was found in this study. However, when looking at specific cancers, maternal overweight or obesity was found to be a risk factor for leukemia and, interestingly, a protective factor for testicular cancer in offspring. In 2010, a meta-analysis performed by Alam et al. suggested that higher maternal weight did not increase testicular cancer risk in offspring, which is inconsistent with our findings [24]. Furthermore, a meta-analysis of 34 studies published in 2022 found pregnancy BMI was positively associated with leukemia risk in offspring (odds ratio [OR] per 5-unit BMI increase = 1.07, 95% CI: 1.04–1.1), which is consistent with our study [47]. In comparison, our meta-analysis not only included pediatric cancer but also cancer in adults, which is thought to be more comprehensive. Previous meta-analyses only considered the highest grades of BMI and included studies that reported only maternal weight rather than BMI. Our study excluded studies that reported only maternal weight rather than BMI or that did not meet the BMI classification criteria, added two large sample studies, and focused on the risk of cancer in overweight or obese offspring. Considering that all included studies adjusted for confounders, although the adjusted confounders were slightly different in each study, maternal overweight or obesity may be a true protective factor for testicular cancer in offspring, but the mechanism is unclear at present. Considering the limited number of references included, more caution should be exercised in interpreting these results and drawing conclusions.

With regard to maternal GWG, a statistically significant association between high GWG and the risk of total cancer in offspring was found. However, when stratified by cancer phenotypes, no significant association was found. Considering the relatively small number of studies included for each specific cancer, the possibility cannot be ruled out that the potential significance of the increased cancer risk cannot be identified due to the limited statistical power. Existing research has shown that insufficient or excessive GWG is not only related to adverse pregnancy outcomes but also has a far-reaching impact on the long-term health of offspring. Excessive GWG is positively associated with a higher risk of C-section, macrocephaly, preeclampsia, gestational diabetes, and postpartum weight retention, while inadequate GWG is also related to a higher risk of placental abruption, small gestational age, preterm birth, and low birth weight [48,49,50,51,52,53]. The latest meta-analysis shows that higher GWG significantly increases the risk of insulin resistance, asthma, wheezing, autism spectrum disorders, atopic dermatitis, overweight, and obesity in offspring [48,54,55,56,57,58]. Unfortunately, the current situation of GWG in pregnant women worldwide is still not optimistic. According to the latest data, the prevalence of GWG above and below the 2009 IOM guidelines was 39.4% and 27.8%, respectively, and only 32.8% of women met the IOM recommendations [59]. Pregnant women are advised to control pregnancy weight gain through a healthy diet and physical activity [60]. It is worth noting that our study included only three studies that classified GWG according to 2009 IOM guidelines. The 2009 IOM guidelines are more appropriate to classify GWG since it takes maternal prepregnancy BMI into account. More studies that classified GWG according to 2009 IOM guidelines are needed in the future to further demonstrate the potential risk of excessive GWG on cancer risk in offspring.

The advantage of our study is the comprehensive search and analysis of all available relevant literature. Before this study, only a meta-analysis published in 2010 assessed the association between maternal prepregnancy BMI and testicular cancer in offspring. Our meta-analysis considered not only maternal BMI, but also the GWG on offspring cancer, and it focused on total cancer and specific cancers. Compared with the previous meta-analysis, our research sample size (8,310,695 participants) is larger, and the analysis is more detailed and comprehensive.

One limitation of our meta-analysis is that no source of heterogeneity was found. We performed a subgroup analysis of factors such as geographic region, study design, study population, ascertainment of maternal weight, whether confounding factors were adjusted, and whether GWG was classified according to 2009 IOM guidelines. Unfortunately, no intergroup differences were found. Due to the limited information provided by the original literature, we cannot further discover the sources of heterogeneity. Although most of the included studies adjusted for confounders, these results are not surprising given that cancer is influenced by many factors, and the confounders adjusted for by different studies differ slightly. Another disadvantage is that due to the limited literature available for each cancer, we only analyzed some specific cancers. Future studies need to refine cancer types to clarify the impact of GWG on different cancers. Moreover, although every effort has been made to minimize the possible deviations in the specific retrieval of major databases, there may still be some unidentified literature. Fortunately, as Begg’s test showed, none of our results found publication bias.

## Figures and Tables

**Table 1:** Selected characteristics of twenty-one studies.

First Author and Year	Study Design	Geographic Region	Sample Size	Study Population (Age)	Ascertainment of Maternal Weight	Maternal Weight Categories	Outcomes Reported	Confounds Adjusted	Quality Assessment
Fu [13] 2021	cohort study	America	5845	male adults (59.8 ± 6.6 y)	self-reported	maternal pre-pregnancy BMI; GWG	overall cancer	age, time period, race, family history of cancer, maternal education, and paternal education	8
Schmid [33] 2020	cohort study	America	35,133	female adults (25–42 y)	self-reported	maternal pre-pregnancy BMI; GWG	breast cancer	age, race, family history of breast cancer, smoking during pregnancy, weight gain during pregnancy, pre-pregnancy BMI, adult caloric intake, adult alcohol intake, adult smoking, and adult BMI	7
Kessous [14] 2020	cohort study	Israel	241,273	children (<18 y)	medical record	maternal pre-pregnancy BMI	overall cancer, lymphoma, leukemia, brain cancer	maternal age, diabetes mellitus, hypertensive disorders, preterm delivery, type of delivery, and fetuses that are large for gestational age	7
Stacy [15] 2019	cohort study	America	1,827,875	children (<14 y)	medical record	maternal pre-pregnancy BMI; GWG	overall cancer, leukemia	maternal age and race	8
Bailey [21] 2017	case-control study	France	3612	children (<15 y)	self-reported	maternal pre-pregnancy BMI; GWG	brain cencer	sex and age	8
Contreras [16] 2016	case-control study	America	281,296	children (<6 y)	medical record	maternal pre-pregnancy BMI; GWG	leukemia; astrocytomas; intracranial and intraspinal embryonal brain tumors; germ cell tumors; hepatoblastoma; neuroblastoma; retinoblastoma; rhabdomyosarcoma; Wilms’ tumor	year of birth, maternal/paternal race/ethnicity, and maternal age	9
Petridou [34] 2015	cohort study	Sweden	3,444,136	children (<14 y)	medical record	maternal BMI	Hodgkin lymphoma; non-Hodgkin lymphoma	sex, maternal education and age, gestational age, and birth order of the index infant	9
Greenop [20] 2014	case-control study	Australia	1398	children (<14 y)	self-reported	maternal pre-pregnancy BMI; GWG	brain tumors	matching variables, child’s year of birth group, maternal age group, child sethnicity, and maternal pre-pregnancy folate supplementation	8
Musselman [23] 2013	case-control study	America	770	children (<6 y)	self-reported	maternal pre-pregnancy BMI	hepatoblastoma	NA	6
Stephansson [35] 2011	case-control study	Nordic countries (Norway, Sweden, Finland, and Denmark)	1672	children (<15 y)	medical record	maternal BMI	testicular germ-cell cancer	birth weight, gestational age, parity, and maternal age	9
Källén [36] 2010	case-control study	Sweden	2,424,336	children and young adults	medical record	maternal BMI	overall cancer	birth, number of previous miscarriages, and years of unwanted childlessness	9
McLaughlin [37] 2009	cohort study	America	12,539	children (1 month–14 y)	medical record	GWG	neuroblastoma	NA	6
Johnson [39] 2008	cohort study	America	9397	Children (28 day–14 y)	medical record	GWG	leukemia	birth year	7
Pettersson [38] 2008	case-control study	Sweden	1154	young adults or adults (age ≥ 15)	medical record	maternal pre-pregnancy BMI; GWG	testicular germ-cell cancer	maternal age at pregnancy and birth order	8
Spector [22] 2007	case-control study	America	495	children (<1 y)	self-reported	maternal pre-pregnancy BMI; GWG	leukemia	sex, race, and maternal education	7
Sonke [40] 2007	case-control study	America	230	adults (18–50 y)	self-reported	maternal pre-pregnancy BMI	testicular germ-cell cancer	mother’s race, education, and body mass index; son’s birth weight, age, and history of cryptorchidism; nausea during pregnancy, and length of pregnancy	7
McLaughlin [41](A) 2006	cohort study	America	10,756	children (<10 y)	medical record	GWG	leukemia	birth year, gender, race and ethnicity, maternal age, gestational age, and birth weight	8
McLaughlin [42](B) 2006	cohort study	America	6114	children (1 month–5 y)	medical record	maternal pre-pregnancy BMI; GWG	hepatoblastoma	birth year and birth weight	7
Coupland [43] 2004	case-control study	The United Kingdom	851	young adults or adults (15–49 y)	self-reported	maternal BMI at index pregnancy	testicular germ-cell cancer	age, region, son’s social class, undescended testis or inguinal hernia before 15 years of age, and maternal age at index pregnancy	7
Weir [44] 2000	case-control study	Canada	867	young adults or adults (16–59 y)	self-reported	maternal BMI at index pregnancy	testicular germ-cell cancer	age	7
Sanderson [45] 1998	case-control study	America	946	female adults (<45 y)	self-reported	maternal pre-pregnancy BMI; GWG	breast cancer	NA	7
Murphy [46] 2022	cohort study	America	18,751	adults (18–56 y)	self-reported	maternal BMI	colorectal cancer	race/ethnicity, gestational age, and maternal BMI (rate of early weight gain); race/ethnicity, gestational age, maternal BMI, and rate of early weight gain (total weight gain); and race/ethnicity, gestational age, maternal BMI, rate of early weight gain, and total weight gain (birth weight)	8

GWG, gestational weight gain.

**Table 2:** Subgroup analyses for the association between maternal BMI and risk of cancer in offspring.

Variable	No. of Studies	RR (95% CI)	I^2^ (%)	*p* Value for Heterogeneity	Test for Subgroup Differences		
					χ^2^	*p*	I^2^ (%)
**Maternal underweight and offspring cancer**							
**Geographic region**					1.53	0.220	35
America	8	1.10 (0.99, 1.22)	0	0.890			
Non-America	7	0.98 (0.84, 1.14)	12	0.340			
**Study design**					1.43	0.230	30
Cohort study	5	1.10 (0.98, 1.23)	0	0.810			
Case-control study	10	1.00 (0.89, 1.12)	0	0.470			
**Study population**					0.01	0.910	0
Children	8	1.04 (0.93, 1.16)	0	0.590			
Adults	7	1.05 (0.93, 1.18)	1	0.420			
**Ascertainment of maternal weight**					1.67	0.200	40
Self-reported	9	1.10 (0.98, 1.23)	0	0.960			
Medical record	6	0.96 (0.81, 1.14)	36	0.160			
**Whether confounding factors were adjusted**					0.00	0.950	0
Yes	13	1.04 (0.96, 1.14)	0	0.610			
No	2	1.03 (0.67, 1.58)	36	0.210			
**Maternal BMI**					0.10	0.750	0
Overweight	9	1.08 (0.98, 1.19)	39	0.110			
Obese	10	1.05 (0.94, 1.18)	29	0.180			
**Maternal Overweight/obesity and offspring cancer**							
**Geographic region**					2.85	0.090	65
America	10	1.14 (1.02, 1.28)	51	0.030			
Non-America	9	0.98 (0.86, 1.12)	32	0.160			
**Study design**					2.94	0.090	66
Cohort study	7	1.24 (1.02, 1.51)	50	0.080			
Case-control study	12	1.03 (0.95, 1.12)	15	0.290			
**Study population**					0.33	0.570	0
Children	11	1.08 (1.00, 1.18)	42	0.070			
Adults	8	1.01 (0.81, 1.26)	53	0.040			
**Ascertainment of maternal weight**					0.02	0.900	0
Self-reported	11	1.06 (0.90, 1.24)	49	0.030			
Medical record	8	1.07 (0.98, 1.17)	45	0.080			
**Whether confounding factors were adjusted**					1.01	0.310	1.2
Yes	17	1.06 (0.97, 1.16)	48	0.010			
No	2	1.21 (0.95, 1.53)	0	0.530			

Risk estimates between maternal underweight and the risk of specific cancers including testicular germ-cell cancer, brain cancer, hepatoblastoma, leukemia, and breast cancer are summarized in Supplemental Figure S1. The overall analysis suggested that maternal underweight was not associated with the risk of testicular germ-cell cancer (OR: 1.02, 95% CI: 0.72–1.44), brain cancer (OR: 1.20, 95% CI: 0.81–1.78), hepatoblastoma (OR: 0.86, 95% CI: 0.48–1.52), leukemia (OR: 0.94, 95% CI: 0.52–1.72), or breast cancer (OR: 1.10, 95% CI: 0.91–1.32) in offspring.

**Table 3:** Subgroup analyses for the association between GWG and risk of cancer in offspring.

Variable	No. of Studies	OR (95% CI)	I^2^ (%)	*p* Value for Heterogeneity	Test for Subgroup Differences		
					χ^2^	*p*	I^2^ (%)
**Maternal low GWG and offspring cancer**							
**Geographic region**					3.20	0.070	69
America	9	1.00 (0.92, 1.09)	25	0.220			
Non-America	3	1.33 (0.99, 1.78)	53	0.120			
**Study design**					0.02	0.880	0
Cohort study	6	1.04 (0.96, 1.12)	0	0.570			
Case-cohort study	6	1.06 (0.84, 1.34)	71	0.004			
**Study population**					1.31	0.250	23
Children	8	1.11 (0.98, 1.27)	47	0.070			
Adults	4	0.98 (0.82, 1.17)	57	0.070			
**Ascertainment of maternal weight**					0.02	0.900	0
Self-reported	6	1.05 (0.86, 1.27)	69	0.007			
Medical record	6	1.03 (0.95, 1.13)	3	0.400			
**Whether confounding factors were adjusted**					0.40	0.530	0
Yes	10	1.07 (0.98, 1.18)	36	0.120			
No	2	0.90 (0.53, 1.52)	81	0.020			
**Whether GWG was classified according to 2009 IOM guidelines**					0.67	0.410	0
Yes	3	1.20 (0.84,1.70)	80	0.007			
No	9	1.03 (0.93,1.13)	27	0.210			
**Maternal high GWG and offspring cancer**							
**Geographic region**					0.00	0.970	0
America	10	1.10 (1.01, 1.19)	42	0.080			
Non-America	3	1.10 (0.78, 1.56)	67	0.050			
**Study design**					1.32	0.250	24
Cohort study	7	1.14 (1.04, 1.25)	30	0.200			
Case-control study	6	1.02 (0.87, 1.21)	44	0.110			
**Study population**					0.39	0.530	0
Children	9	1.08 (0.97, 1.20)	51	0.040			
Adults	4	1.14 (1.00, 1.30)	20	0.290			
**Ascertainment of maternal weight**					0.00	0.950	0
Self-reported	6	1.10 (0.94, 1.28)	49	0.080			
Medical record	7	1.09 (0.98, 1.21)	46	0.090			
**Whether confounding factors were adjusted**					1.19	0.270	16
Yes	11	1.11 (1.02, 1.22)	48	0.040			
No	2	0.95 (0.73, 1.24)	23	0.250			
**Whether GWG was classified according to 2009 IOM guidelines**					0.64	0.420	0
Yes	3	1.01 (0.79,1.30)	66	0.050			
No	10	1.13 (1.04,1.23)	23	0.230			

GWG, gestational weight gain; IOM, the Institute of Medicine.

## Data Availability

The data behind the article can be found in the text and online supplementary material.

## Supplementary Materials

The following supporting information can be downloaded at: https://www.mdpi.com/article/10.3390/nu15071601/s1, Table S1: Sensitivity analyses results. Figure S1: Forest plot of the association between maternal underweight and the risk of specific cancer in offspring. Figure S2: Forest plot of the association between maternal overweight/obesity and the risk of specific cancer in offspring. Figure S3: Forest plot of the association between maternal low GWG and the risk of specific cancer in offspring. Figure S4: Forest plot of the association between maternal high GWG and the risk of specific cancer in offspring.
