# Supplementary material for: Maternal Body Mass Index, Gestational Weight Gain, and Risk of Cancer in Offspring: A Systematic Review and Meta-Analysis
Source: Nutrients. 2023 Mar 25;15(7):1601. doi: 10.3390/nu15071601 (PMC10096488; doi:10.3390/nu15071601)
Supplement: Supplementary file 1 [file nutrients-15-01601-s001.zip › nutrients-2268579-supplementary.pdf]

Supplemental Table S1. Sensitivity analyses results.

Study Omitted	OR	LCI	UCI	p	I^2 (%)
**Underweight (OR: 1.05, 95% CI: 0.966, 1.13; p =0.264)**					
Bailey 2017	1.05	0.96	1.13	0.29	0
Contreras 2016	1.05	0.97	1.14	0.24	0
Coupland 2004	1.04	0.96	1.13	0.36	0
Fu 2021	1.04	0.95	1.14	0.36	0
Greenop 2014	1.04	0.96	1.13	0.31	0
Källén 2010	1.07	0.98	1.18	0.13	0
McLaughlin (Hepatoblastoma) 2006	1.05	0.96	1.13	0.28	0
Musselman 2013	1.05	0.97	1.14	0.22	0
Petridou 2015	1.05	0.97	1.14	0.24	0
Pettersson 2008	1.06	0.98	1.15	0.14	0
Sanderson 1998	1.04	0.96	1.13	0.37	0
Schmid 2020	1.04	0.96	1.14	0.32	0
Sonke 2007	1.05	0.96	1.13	0.28	0
Stacy 2019	1.02	0.93	1.12	0.65	0
Weir 2000	1.05	0.97	1.14	0.23	0
**Overweight/obesity (OR: 1.07, 95% CI: 0.99, 1.16; p =0.100)**					
Bailey 2017	1.08	0.99	1.18	0.06	31
Contreras 2016	1.07	0.97	1.19	0.15	32
Coupland 2004	1.08	0.99	1.17	0.06	30
Fu 2021	1.08	0.99	1.17	0.10	32
Greenop 2014	1.07	0.98	1.17	0.11	32
Kessous 2020	1.06	0.98	1.15	0.16	28
Källén 2010	1.08	0.98	1.19	0.11	32
McLaughlin (Hepatoblastoma) 2006	1.06	0.98	1.14	0.14	27
Murphy 2022	1.05	0.98	1.13	0.16	23
Musselman 2013	1.06	0.97	1.15	0.18	31
Petridou 2015	1.07	0.98	1.17	0.13	32
Pettersson 2008	1.08	0.99	1.17	0.09	32
Sanderson 1998	1.07	0.98	1.17	0.12	32
Schmid 2020	1.08	0.98	1.17	0.10	32
Sonke 2007	1.07	0.99	1.17	0.09	32
Spector 2007	1.06	0.98	1.15	0.17	31
Stacy 2019	1.08	0.97	1.20	0.14	32
Stephansson 2011	1.07	0.99	1.17	0.08	30
Weir 2000	1.08	0.99	1.17	0.07	30
**Low GWG (OR: 1.06, 95% CI: 0.96, 1.17; p =0.270)**					
Bailey 2017	1.06	0.95	1.18	0.312	52
Contreras 2016	1.08	0.96	1.21	0.185	50
Fu 2021	1.06	0.95	1.19	0.319	52

Greenop 2014	1.02	0.94	1.10	0.672	16
McLaughlin 2009	1.05	0.95	1.17	0.355	51
McLaughlin (ALL) 2006	1.04	0.94	1.15	0.447	46
McLaughlin (Hepatoblastoma) 2006	1.06	0.95	1.17	0.292	52
Pettersson 2008	1.05	0.95	1.17	0.356	51
Sanderson 1998	1.08	0.99	1.17	0.103	32
Schmid 2020	1.08	0.96	1.21	0.226	52
Spector 2007	1.06	0.96	1.18	0.248	52
Stacy 2019	1.08	0.95	1.22	0.245	52
**High GWG (OR: 1.10, 95% CI: 1.01, 1.19; p =0.040)**					
Bailey 2017	1.11	1.03	1.21	0.010	38
Contreras 2016	1.12	1.02	1.22	0.014	38
Fu 2021	1.08	0.99	1.18	0.093	44
Greenop 2014	1.08	0.99	1.18	0.068	43
Johnson 2008	1.08	0.99	1.18	0.080	44
McLaughlin 2009	1.10	1.00	1.20	0.044	49
McLaughlin (ALL) 2006	1.08	0.99	1.18	0.091	42
McLaughlin (Hepatoblastoma) 2006	1.11	1.02	1.20	0.013	39
Pettersson 2008	1.09	1.00	1.19	0.057	48
Sanderson 1998	1.11	1.02	1.21	0.016	43
Schmid 2020	1.09	0.99	1.20	0.081	48
Spector 2007	1.09	1.00	1.19	0.052	49
Stacy 2019	1.10	0.99	1.22	0.079	49

LCI, lower 95% confidence interval; OR, odds ratio; UCI, upper 95% confidence interval; GWG, gestational weight gain.

Study or Subgroup	log[Odds Ratio]	SE	Weight	Odds Ratio IV, Random, 95% CI
**testicular germ-cell cancer**				
Contreras 2016	0.7608	0.4825	10.3%	2.14 [0.83, 5.51]
Coupland 2004	0.158	0.1687	32.2%	1.17 [0.84, 1.63]
Pettersson 2008	-0.462	0.2408	24.6%	0.63 [0.39, 1.01]
Sonke 2007	0.2624	0.4905	10.0%	1.30 [0.50, 3.40]
Weir 2000	-0.1054	0.2606	22.8%	0.90 [0.54, 1.50]
**Total (95% CI)**			**100.0%**	**1.02 [0.72, 1.44]**
Heterogeneity: Tau² = 0.07; Chi² = 7.49, df = 4 (P = 0.11); I² = 47%				
Test for overall effect: Z = 0.10 (P = 0.92)				
**brain cancer**				
Bailey 2017	0.0953	0.2221	82.5%	1.10 [0.71, 1.70]
Greenop 2014	0.5878	0.4819	17.5%	1.80 [0.70, 4.63]
**Total (95% CI)**			**100.0%**	**1.20 [0.81, 1.78]**
Heterogeneity: Tau² = 0.00; Chi² = 0.86, df = 1 (P = 0.35); I² = 0%				
Test for overall effect: Z = 0.90 (P = 0.37)				
**hepatoblastoma**				
McLaughlin(B) 2006	0.3365	0.6392	20.7%	1.40 [0.40, 4.90]
Musselman 2013	-0.2821	0.3264	79.3%	0.75 [0.40, 1.43]
**Total (95% CI)**			**100.0%**	**0.86 [0.48, 1.52]**
Heterogeneity: Tau² = 0.00; Chi² = 0.74, df = 1 (P = 0.39); I² = 0%				
Test for overall effect: Z = 0.53 (P = 0.60)				
**leukemia**				
Contreras 2016	-0.478	0.3658	35.0%	0.62 [0.30, 1.27]
Stacy 2019	0.1655	0.098	65.0%	1.18 [0.97, 1.43]
**Total (95% CI)**			**100.0%**	**0.94 [0.52, 1.72]**
Heterogeneity: Tau² = 0.14; Chi² = 2.89, df = 1 (P = 0.09); I² = 65%				
Test for overall effect: Z = 0.19 (P = 0.85)				
**breast cancer**				
Sanderson 1998	0.1823	0.1777	28.4%	1.20 [0.85, 1.70]
Schmid 2020	0.0583	0.1119	71.6%	1.06 [0.85, 1.32]
**Total (95% CI)**			**100.0%**	**1.10 [0.91, 1.32]**
Heterogeneity: Tau² = 0.00; Chi² = 0.35, df = 1 (P = 0.55); I² = 0%				
Test for overall effect: Z = 0.99 (P = 0.32)				

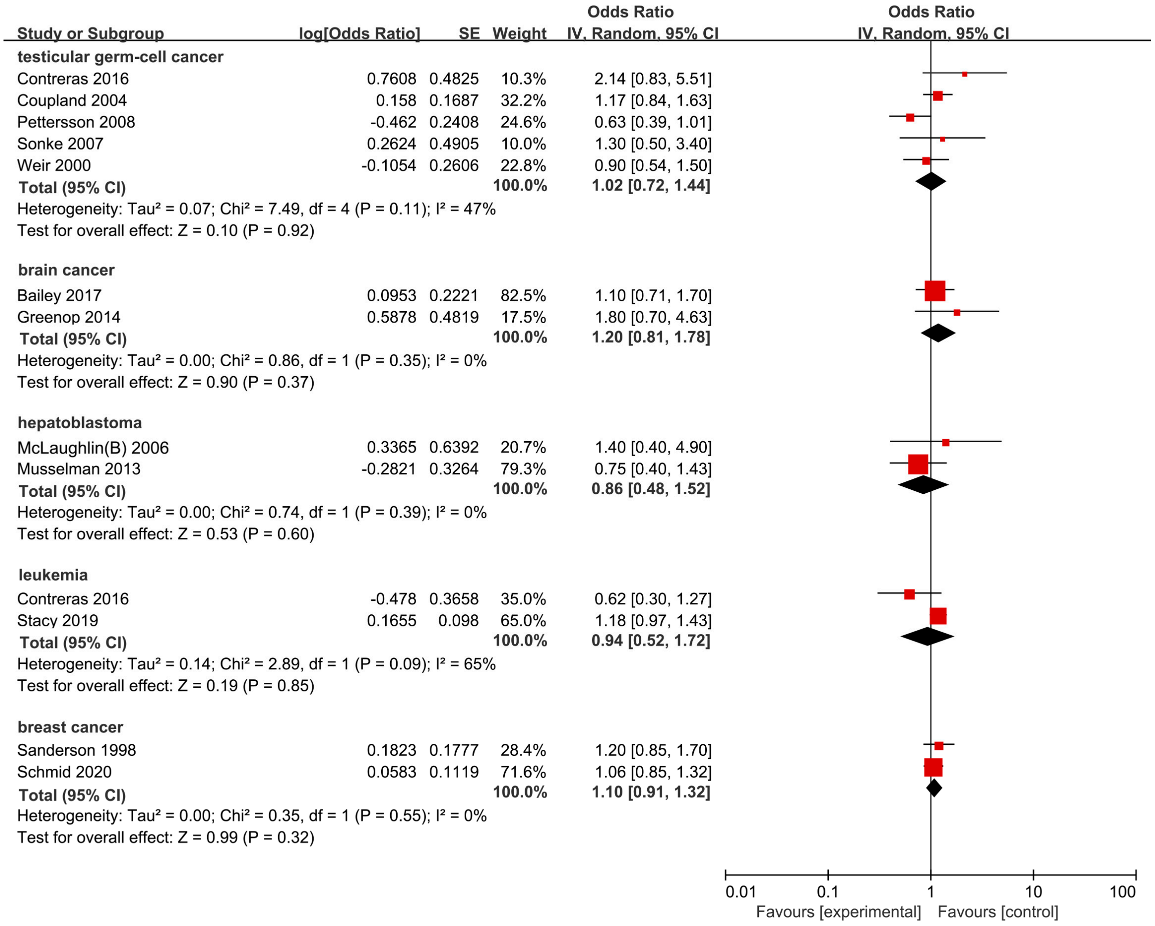

Figure S1: Forest plot of the association between maternal underweight and the risk of specific cancer in offspring.

Study or Subgroup	log[Odds Ratio]	SE	Weight	Odds Ratio IV, Random, 95% CI
**testicular germ-cell cancer**				
Contreras 2016	-0.1165	0.2348	25.5%	0.89 [0.56, 1.41]
Coupland 2004	-0.2744	0.2157	30.2%	0.76 [0.50, 1.16]
Pettersson 2008	-0.1054	0.2741	18.7%	0.90 [0.53, 1.54]
Sonke 2007	-0.5108	0.6143	3.7%	0.60 [0.18, 2.00]
Stephansson 2011	-0.8916	0.648	3.3%	0.41 [0.12, 1.46]
Weir 2000	-0.3567	0.275	18.6%	0.70 [0.41, 1.20]
**Total (95% CI)**			**100.0%**	**0.78 [0.62, 0.99]**
Heterogeneity: Tau² = 0.00; Chi² = 1.92, df = 5 (P = 0.86); I² = 0%				
Test for overall effect: Z = 2.09 (P = 0.04)				
**leukemia**				
Contreras 2016	0.1906	0.0872	31.6%	1.21 [1.02, 1.44]
Kessous 2020	-0.2231	1.0107	0.2%	0.80 [0.11, 5.80]
Spector 2007	0.2927	0.1888	6.7%	1.34 [0.93, 1.94]
Stacy 2019	0.1398	0.0626	61.4%	1.15 [1.02, 1.30]
**Total (95% CI)**			**100.0%**	**1.18 [1.07, 1.30]**
Heterogeneity: Tau² = 0.00; Chi² = 0.85, df = 3 (P = 0.84); I² = 0%				
Test for overall effect: Z = 3.37 (P = 0.0007)				
**brain cancer**				
Bailey 2017	-0.1744	0.1734	31.9%	0.84 [0.60, 1.18]
Contreras 2016	-0.3011	0.216	26.3%	0.74 [0.48, 1.13]
Greenop 2014	0.0392	0.1369	37.3%	1.04 [0.80, 1.36]
Kessous 2020	1.3863	0.7073	4.6%	4.00 [1.00, 16.00]
**Total (95% CI)**			**100.0%**	**0.94 [0.69, 1.29]**
Heterogeneity: Tau² = 0.05; Chi² = 6.38, df = 3 (P = 0.09); I² = 53%				
Test for overall effect: Z = 0.36 (P = 0.72)				
**hepatoblastoma**				
Contreras 2016	-0.1165	0.2163	35.6%	0.89 [0.58, 1.36]
McLaughlin(B) 2006	0.9203	0.3647	22.9%	2.51 [1.23, 5.13]
Musselman 2013	0.2506	0.1571	41.5%	1.28 [0.94, 1.75]
**Total (95% CI)**			**100.0%**	**1.31 [0.83, 2.08]**
Heterogeneity: Tau² = 0.11; Chi² = 6.14, df = 2 (P = 0.05); I² = 67%				
Test for overall effect: Z = 1.16 (P = 0.24)				
**breast cancer**				
Sanderson 1998	0.0953	0.1912	34.6%	1.10 [0.76, 1.60]
Schmid 2020	0.0198	0.1392	65.4%	1.02 [0.78, 1.34]
**Total (95% CI)**			**100.0%**	**1.05 [0.84, 1.31]**
Heterogeneity: Tau² = 0.00; Chi² = 0.10, df = 1 (P = 0.75); I² = 0%				
Test for overall effect: Z = 0.41 (P = 0.68)				

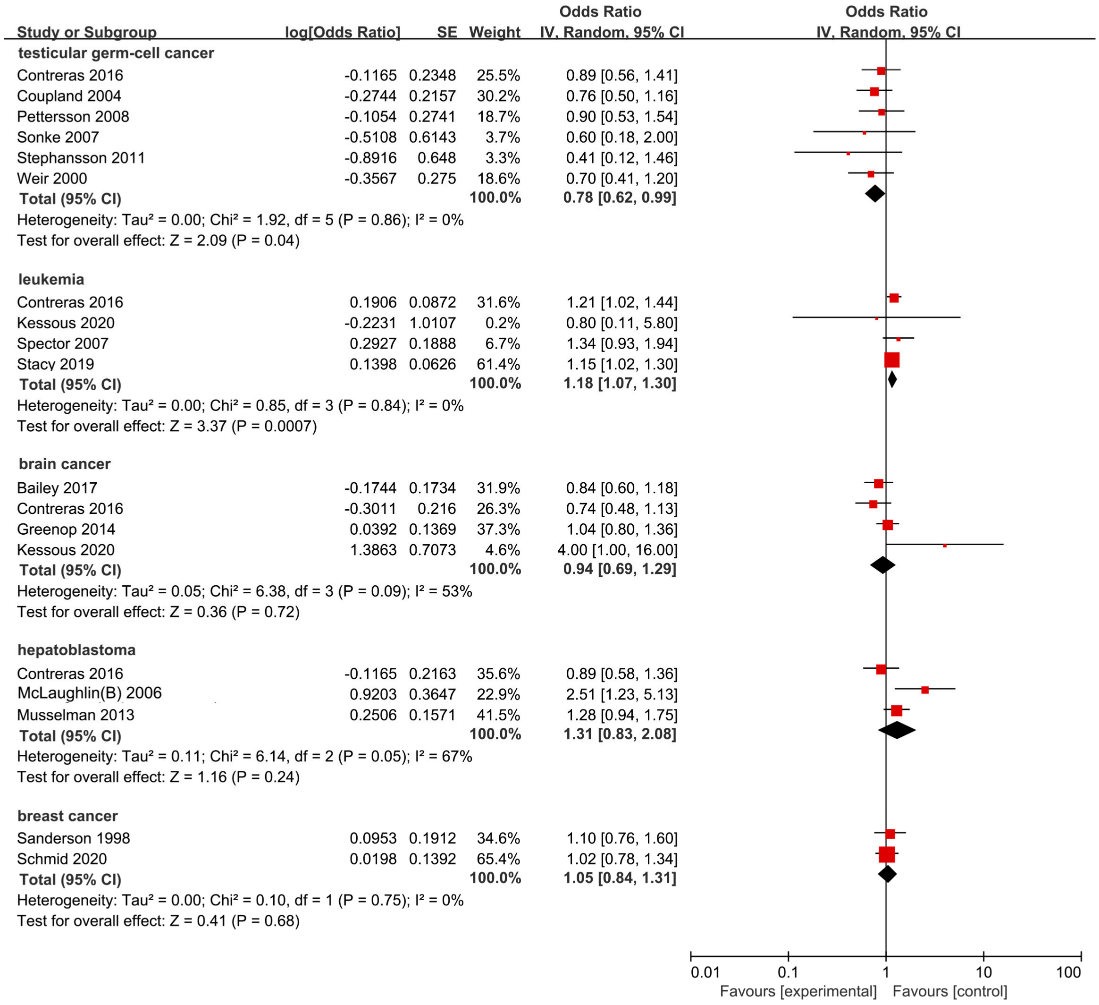

Figure S2: Forest plot of the association between maternal overweight/obesity and the risk of specific cancer in offspring.

Study or Subgroup	log[Odds Ratio]	SE	Weight	Odds Ratio IV, Random, 95% CI
**leukemia**				
Contreras 2016	-0.0726	0.1343	15.6%	0.93 [0.71, 1.21]
McLaughlin(A) 2006	0.2927	0.17	9.9%	1.34 [0.96, 1.87]
Spector 2007	-0.0486	0.2569	4.4%	0.95 [0.58, 1.58]
Stacy 2019	0.0079	0.0571	70.1%	1.01 [0.90, 1.13]
**Total (95% CI)**			**100.0%**	**1.02 [0.92, 1.14]**
Heterogeneity: Tau² = 0.00; Chi² = 3.16, df = 3 (P = 0.37); I² = 5%				
Test for overall effect: Z = 0.39 (P = 0.70)				
**brain cancer**				
Bailey 2017	0.0953	0.1582	40.7%	1.10 [0.81, 1.50]
Contreras 2016	0.0488	0.2917	23.2%	1.05 [0.59, 1.86]
Greenop 2014	0.5878	0.1876	36.1%	1.80 [1.25, 2.60]
**Total (95% CI)**			**100.0%**	**1.30 [0.91, 1.85]**
Heterogeneity: Tau² = 0.05; Chi² = 4.66, df = 2 (P = 0.10); I² = 57%				
Test for overall effect: Z = 1.45 (P = 0.15)				
**breast cancer**				
Sanderson 1998	-0.3668	0.1587	42.1%	0.69 [0.51, 0.95]
Schmid 2020	-0.0101	0.072	57.9%	0.99 [0.86, 1.14]
**Total (95% CI)**			**100.0%**	**0.85 [0.60, 1.20]**
Heterogeneity: Tau² = 0.05; Chi² = 4.19, df = 1 (P = 0.04); I² = 76%				
Test for overall effect: Z = 0.91 (P = 0.36)				
**testicular germ-cell cancer**				
Contreras 2016	0.27	0.3338	25.0%	1.31 [0.68, 2.52]
Pettersson 2008	0.1989	0.1927	75.0%	1.22 [0.84, 1.78]
**Total (95% CI)**			**100.0%**	**1.24 [0.90, 1.72]**
Heterogeneity: Tau² = 0.00; Chi² = 0.03, df = 1 (P = 0.85); I² = 0%				
Test for overall effect: Z = 1.30 (P = 0.19)				
**hepatoblastoma**				
Contreras 2016	-0.1625	0.3596	50.1%	0.85 [0.42, 1.72]
McLaughlin(B) 2006	0.1655	0.3601	49.9%	1.18 [0.58, 2.39]
**Total (95% CI)**			**100.0%**	**1.00 [0.61, 1.65]**
Heterogeneity: Tau² = 0.00; Chi² = 0.42, df = 1 (P = 0.52); I² = 0%				
Test for overall effect: Z = 0.00 (P = 1.00)				
**neuroblastoma**				
Contreras 2016	-0.2231	0.2026	45.8%	0.80 [0.54, 1.19]
McLaughlin 2009	0.1674	0.168	54.2%	1.18 [0.85, 1.64]
**Total (95% CI)**			**100.0%**	**0.99 [0.68, 1.45]**
Heterogeneity: Tau² = 0.04; Chi² = 2.20, df = 1 (P = 0.14); I² = 55%				
Test for overall effect: Z = 0.06 (P = 0.95)				

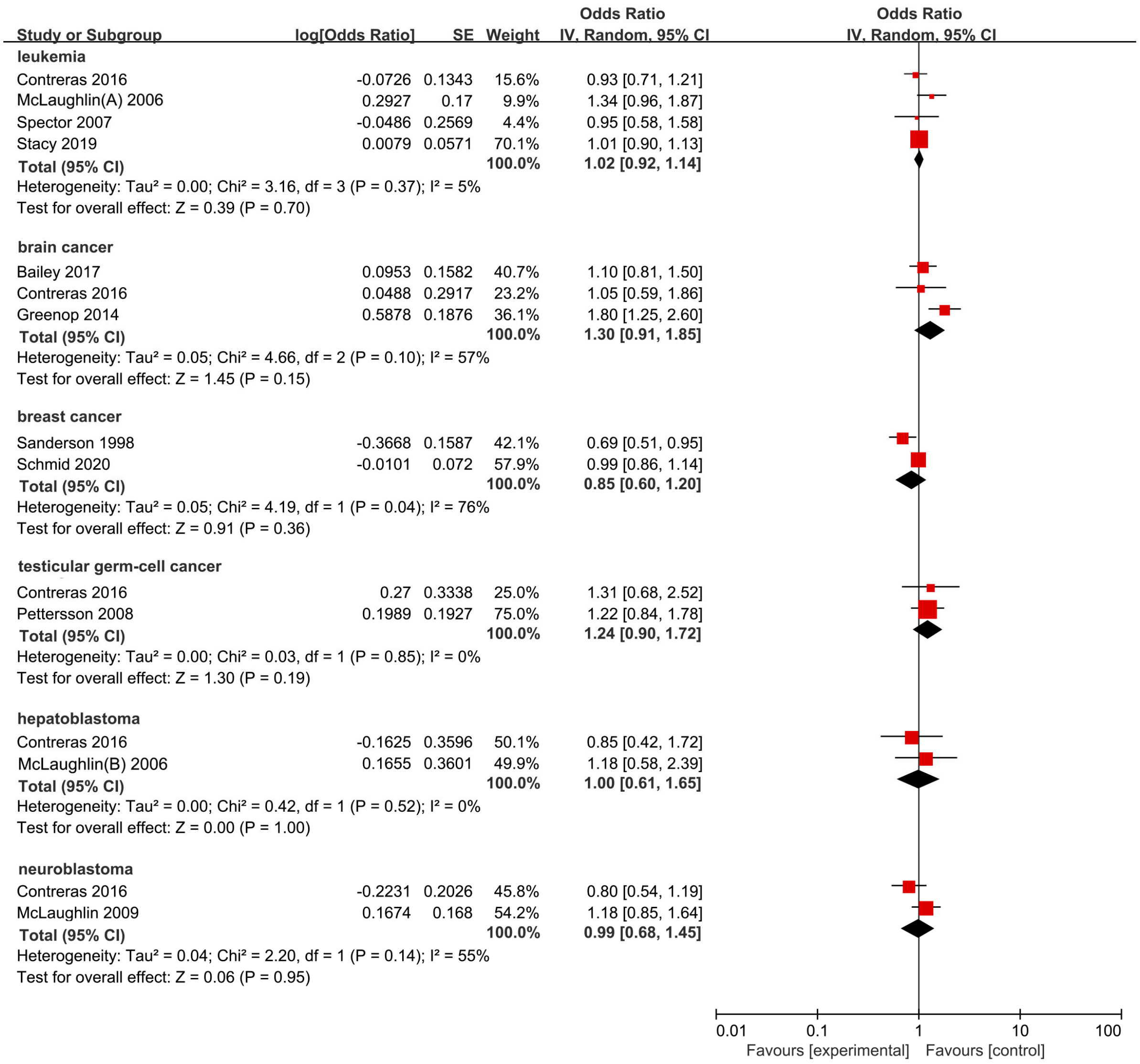

Figure S3: Forest plot of the association between maternal low GWG and the risk of specific cancer in offspring.

Study or Subgroup	log[Odds Ratio]	SE	Weight	Odds Ratio IV, Random, 95% CI
**leukemia**				
Contreras 2016	-0.1508	0.1162	18.9%	0.86 [0.68, 1.08]
Johnson 2008	0.2469	0.1264	17.2%	1.28 [1.00, 1.64]
McLaughlin(A) 2006	0.2469	0.1059	20.8%	1.28 [1.04, 1.58]
Spector 2007	0.1634	0.2068	8.7%	1.18 [0.79, 1.77]
Stacy 2019	0.0712	0.048	34.4%	1.07 [0.98, 1.18]
**Total (95% CI)**			**100.0%**	**1.11 [0.97, 1.27]**
Heterogeneity: Tau² = 0.01; Chi² = 8.31, df = 4 (P = 0.08); I² = 52%				
Test for overall effect: Z = 1.52 (P = 0.13)				
**brain cancer**				
Bailey 2017	-0.2231	0.1625	36.8%	0.80 [0.58, 1.10]
Contreras 2016	-0.4155	0.2744	27.2%	0.66 [0.39, 1.13]
Greenop 2014	0.3365	0.1717	36.0%	1.40 [1.00, 1.96]
**Total (95% CI)**			**100.0%**	**0.93 [0.60, 1.45]**
Heterogeneity: Tau² = 0.11; Chi² = 7.94, df = 2 (P = 0.02); I² = 75%				
Test for overall effect: Z = 0.33 (P = 0.74)				
**breast cancer**				
Sanderson 1998	-0.2324	0.2063	37.3%	0.79 [0.53, 1.19]
Schmid 2020	0.1398	0.0856	62.7%	1.15 [0.97, 1.36]
**Total (95% CI)**			**100.0%**	**1.00 [0.70, 1.42]**
Heterogeneity: Tau² = 0.04; Chi² = 2.78, df = 1 (P = 0.10); I² = 64%				
Test for overall effect: Z = 0.01 (P = 1.00)				
**testicular germ-cell cancer**				
Contreras 2016	0.0677	0.3036	28.1%	1.07 [0.59, 1.94]
Pettersson 2008	0.1989	0.1899	71.9%	1.22 [0.84, 1.77]
**Total (95% CI)**			**100.0%**	**1.18 [0.86, 1.61]**
Heterogeneity: Tau² = 0.00; Chi² = 0.13, df = 1 (P = 0.71); I² = 0%				
Test for overall effect: Z = 1.01 (P = 0.31)				
**hepatoblastoma**				
Contreras 2016	0.2231	0.2791	51.5%	1.25 [0.72, 2.16]
McLaughlin(B) 2006	-0.4943	0.3054	48.5%	0.61 [0.34, 1.11]
**Total (95% CI)**			**100.0%**	**0.88 [0.44, 1.78]**
Heterogeneity: Tau² = 0.17; Chi² = 3.01, df = 1 (P = 0.08); I² = 67%				
Test for overall effect: Z = 0.35 (P = 0.73)				
**neuroblastoma**				
Contreras 2016	0	0.1569	44.1%	1.00 [0.74, 1.36]
McLaughlin 2009	0.0515	0.1394	55.9%	1.05 [0.80, 1.38]
**Total (95% CI)**			**100.0%**	**1.03 [0.84, 1.26]**
Heterogeneity: Tau² = 0.00; Chi² = 0.06, df = 1 (P = 0.81); I² = 0%				
Test for overall effect: Z = 0.28 (P = 0.78)				

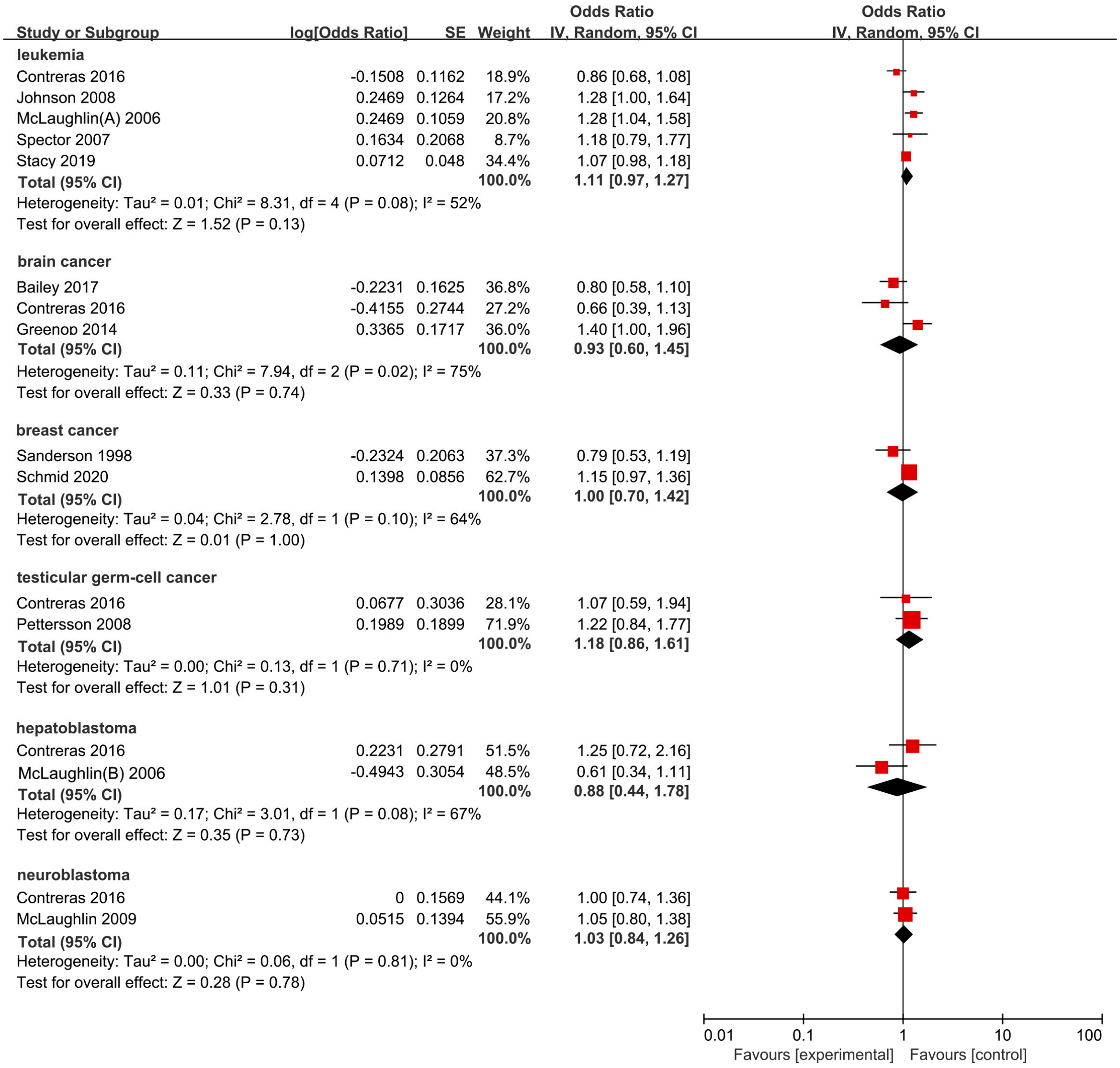

Figure S4: Forest plot of the association between maternal high GWG and the risk of specific cancer in offspring.
